# Point‐of‐care hepatitis C testing and treatment strategy for people attending harm reduction and addiction centres for hepatitis C elimination

**DOI:** 10.1111/jvh.13634

**Published:** 2021-11-29

**Authors:** Xavier Forns, Joan Colom, Montse García‐Retortillo, Joan Carles Quer, Sabela Lens, Elisa Martró, Raquel Domínguez‐Hernández, Miguel Ángel Casado, María Buti

**Affiliations:** ^1^ Liver Unit Hospital Clínic de Barcelona IDIBAPS University of Barcelona Barcelona Spain; ^2^ CIBERehd Instituto Carlos III Barcelona Spain; ^3^ Programme for Prevention, Control and Treatment of HIV, STIs and Viral Hepatitis Programme on Substance Abuse Agency of Public Health of Catalonia Barcelona Spain; ^4^ Department of Gastroenterology Liver Section, Hospital del Mar Barcelona Spain; ^5^ Gastroenterology Department University Hospital Joan XXIII Tarragona Spain; ^6^ Microbiology Department Laboratori Clínic Metropolitana Nord. Hospital Universitari Germans Trias i Pujol. Institut d'Investigació en Ciències de la Salut Germans Trias i Pujol (IGTP) Badalona Spain; ^7^ Biomedical Research Networking Centre in Epidemiology and Public Health (CIBERESP) Instituto de Salud Carlos III. Madrid Spain; ^8^ Pharmacoeconomics & Outcomes Research Iberia (PORIB) Madrid Spain; ^9^ Liver Unit Internal Medicine Department Hospital Universitari Vall d'Hebron Barcelona Spain

**Keywords:** addiction centres, cascade of care, drug users, harm reduction services, hepatitis C virus, linkage‐to‐care, test and treat

## Abstract

According to WHO goals, the elimination of Hepatitis C Virus (HCV) by 2030 requires enhancing and simplifying HCV testing. Our aim was to create a model to assess and compare different strategies for HCV testing, linkage to care and treatment among people who access harm reduction centres (HRC) and Addiction Centres in Catalonia. A decision tree model was designed to evaluate two strategies: Hepatitis C Point‐of‐care (POC) “test and treat”, at the community versus standard‐of‐care (SOC), in which HCV testing was performed at community and therapy at the hospital. Both strategies were assessed separately in HRCs (6,878 users) and Addiction Centres (13,778 users). with a time horizon of 18 months. Healthcare outcomes were HCV testing, linkage to care, treatment outcomes and reinfection rate. HCV testing was performed in 3,178 (46%) of the HRC users. Compared with SOC, POC increased access to treatment by 57% (63% vs. 6%). SVR rates were 64% in POC vs. 23% in SOC. Reinfection rates were 21% with POC compared to 24% with SOC. With POC, losses to follow‐up were reduced by 41%. In the Addiction Centres, 12,566 users (91%) were screened using the two strategies. Compared to the SOC, POC increased access to treatment and linkage to care by 19% along with SVR at the same rate. Reinfection rates decreased by 6%. Thus, the implementation of a POC “test and treat” strategy at HRCs and Addiction Centres has shown to be an effective public health strategy to help eliminating HCV in accordance with WHO goal.

## 1 INTRODUCTION

Hepatitis C virus (HCV) causes significant health and economic burden and reduces the quality of life of the patients. To reach the goal of eliminating hepatitis C by 2030 set by the World Health Organization (WHO),^1^ a public health approach is needed to develop strategies aimed at screening and simplifying HCV infection diagnosis and facilitating early linkage to care and treatment, especially among drug users.

Drug users have a high risk of contracting HCV and encounter many barriers to access diagnosis and treatment within the conventional health system.^1^ In Catalonia, there are different services and centres, including harm reduction centres (HRCs) aimed at serving active drug users who do not want or cannot initiate opioid substitution therapy (OST) and addiction centres (ACs) specialized in the treatment of all drug addictions offering outpatient therapeutic support. In both types of centres, the prevalence of undiagnosed active HCV infection is high, which is a notable obstacle to the administration of effective treatment.

Currently, there are point‐of‐care tests for the determination of HCV viraemia that are easy to use and facilitate infection screening in outpatient care settings, avoiding the need for hospital referral in cases without confirmed HCV infection. Indeed, the decentralization of diagnosis and treatment dispensation based on ‘test and treat’ strategies^2^ would help improve continuity of care in at‐risk populations, such as users who attend HRCs and ACs, and would eventually help achieve hepatitis C elimination.

The objective of our analysis was to perform modelling to simulate and compare different testing, linkage‐to‐care and antiviral treatment strategies for viraemic HCV infection in users of drug addiction care centres in Catalonia.

## 2 METHODS

Two strategies were analysed separately for HRCs and ACs: (a) ‘test and treat at the point‐of‐care’ (POC strategy) and (b) ‘standard of care’ (SOC strategy).

To estimate and simulate the representative cascade of care, a theoretical model was designed with a different decision tree for each strategy, based on data retrieved from clinical practice and the opinion of a multidisciplinary panel of experts. The models integrated the steps necessary for the management of individuals with HCV: testing and diagnosis of viraemic infection, linkage to care, treatment initiation, and evaluation of sustained virologic response (SVR) and reinfection. With the POC strategy, HCV RNA‐positive users received treatment at the same centre (HRCs or ACs). In contrast, with the SOC strategy, HCV RNA‐positive users were referred to the hospital for evaluation, treatment initiation and subsequent follow‐up. Patients were categorized into SVR, no response or lost to follow‐up (the user did not attend medical visits). Reinfection was evaluated only in users achieving SVR. The time horizon of the analysis was 18 months (Figure S1).

The target population was defined as the total number of new users annually attending the HRCs (6,878 users) and the ACs (13,778 users) based on the drug information system of Catalonia.^3^ The same percentages of users screened and the number of HCV RNA‐positive patients were assumed for both strategies, according to the type of centre. A literature search was performed to obtain data to develop the decision tree (percentages of users screened, HCV RNA positivity, referral to hospital, treatment initiation, SVR rates in an intention to treat (ITT) analysis, no response and reinfection). The expert panel provided an estimation for data in which the information was not available.^4^, ^5^, ^6^, ^7^, ^8^, ^9^ (Table S1 and Figure S1).

For each type of centre, the results of the POC and SOC strategies were compared in terms of linkage to care, access to treatment, the SVR at the 12‐week follow‐up after the end of antiviral therapy (SVR12) and the reinfection rate.

## 3 RESULTS

### 3.1 Harm reduction centres

**FIGURE 1:**
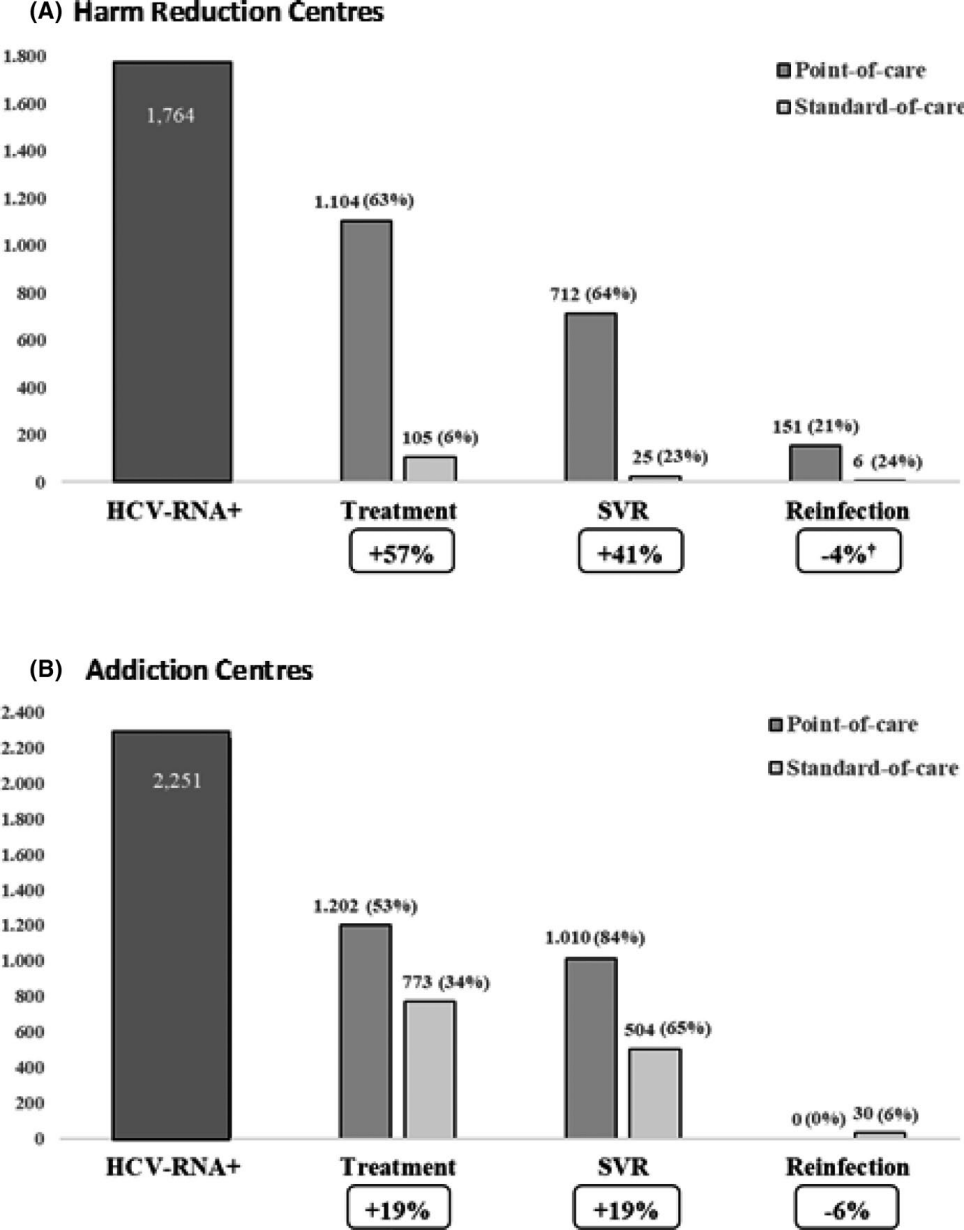
Results of the cascade of testing, linkage to care and treatment outcomes in Harm Reduction Centers (A) and in Addiction Centers (B) comparing Point‐of‐Care and Standard‐of‐Care. ^†^When comparing the number of reinfections in both strategies with respect to patients whose response was previously evaluated (SVR), the point‐of‐care strategy slightly decreased the number of cases [21% (151/712) vs. 25% (6/25)]. The number of treated users was calculated over the total number of viraemic infections and SVR among those who started and continued treatment. SVR, Sustained virologic response

In the simulation, among 6,878 users attending HRCs in both strategies, 3,178 (46%) accepted testing, and 1,764 (56%) were HCV RNA positive. POC strategy compared to the SOC strategy increased access to treatment by 57% (63% vs. 6%) over the total number of viraemic patients and increased the SVR rates by 41% (64% vs. 23%). The remaining 31% (339 users) in the POC group and 72% (75 users) in the SOC group were considered as lost to follow‐up. The incidence of reinfection was 4% lower in the POC group (Figure 1). Of the total viraemic patients, the POC strategy avoided 42% of the viraemic patients from being lost to follow‐up or being untreated over the study period.

### 3.2 Addiction centres

Among users attending ACs (13,778 users), 12,566 were screened for HCV RNA, and 2,251 active infections were detected with both strategies. Compared with the SOC strategy, POC showed a 19% increase in access to treatment and in the response rate. Of the remaining patients, 155 (13%) vs. 235 (30%) were lost to follow‐up; thus, the POC strategy decreased the number of patients lost to follow‐up by 17%. In the POC strategy, none of the patients developed reinfection, while in the SOC strategy group, reinfection occurred in 6% (Figure 1). Therefore, the POC strategy decreased the proportion of patients lost to follow‐up or not treated/not linked to the health system by 23% throughout the analysis period.

## 4 DISCUSSION

Antibody tests for HCV have been available in a number of drug addiction care centres for years in Catalonia.^10^ However, this strategy has not been demonstrated to guarantee linkage to specialized care and treatment; thus, it is necessary to promote effective strategies in this area. For this reason, our analysis compared two diagnostic and treatment strategies for viraemic HCV users attending HRCs and ACs.

The main difference between the two strategies consisted of the need for referral to hospitals or not. To compare the strategies, a model using a decision tree was constructed that allowed the simulation of the different interventions and the extrapolation of the results. It should be noted that the data included in the analysis were obtained from a large group of representative centres involved in public health in the area. For the modelling, the data were extrapolated to the entire population attending HRCs and ACs in Catalonia. Using data from the same community gives consistency to the analysis and reinforces its results.

The results of the analysis showed clear differences between the two strategies. The traditional referral route, in which the initiation of treatment requires additional follow‐up visits, causes users to disengage from medical care and to finally not access treatment.^2^ Our results showed that compared to the SOC strategy, making tests and treatment available in the same centres that drug users usually attend (the POC strategy) decreases losses to follow‐up and increases the number of users who start antiviral treatment and achieve SVR. In addition, the POC strategy slightly decreased the number of reinfections, and although in the HRCs, the reinfection rate remained above 20%. This fact could be explained because, regardless of the strategy implemented, the target population is people who present risk practices for HCV transmission. In evaluating these results, we must consider that they are based on a theoretical model that performs a simplified simulation of the two examined strategies. Nevertheless, this model was validated by a panel of experts, and therefore, its results should be considered valid estimates that can aid in decision‐making in clinical practice and in public health policies.

On the other hand, the contribution of the POC strategy in the treatment of hepatitis C can also be evaluated based on our results. The use of a *‘*test and treat’ approach in POCs helps ensure the immediate transition from diagnosis to treatment and, therefore, helps avoid viraemic users being lost to follow‐up. Consequently, a greater number of users can access effective treatments that result in a cure in most cases and prevent ongoing transmission of the virus, assuming it has a relevant impact on the prevention of the disease.

This analysis has some limitations. The first is related to the assumption considering the same percentages of users with viral load in both strategies. HCV RNA cannot be currently evaluated on‐site in some centres, so in these cases, a standard referral to the hospital is made when a positive HCV serology is found. This strategy assumes a significant loss of users who do not follow through with confirmation tests^10^ and would affect the SOC strategy. In this sense, point of care is being promoted in Catalonia,^10^ and the HCV RNA data in our analysis are the actual results for this strategy in the field at HRCs and ACs. If the standard referral to the hospital had been considered in the SOC strategy, the results of the analysis would have favoured the POC strategy even more. The second limitation is that there is a percentage of users in whom reinfection may occur before the treatment outcome is evaluated (SVR12). In these cases, there is no clinical diagnosis of virologic response, but it is possible to detect genetic changes in the virus before and after treatment by phylogenetic analysis, indicating reinfection. In our analysis, reinfection was assumed only in those cases that were HCV RNA positive beyond the first 12 weeks after the end of treatment, when the cure was confirmed. Therefore, the reinfection rate may have been underestimated.

In conclusion, the implementation of the ‘test and treat’ POC strategy in HRCs and ACs would improve linkage to healthcare and access to treatment and would reduce reinfections, making it an effective public health strategy in Catalonia. Although the detection of viraemia, including reinfections in HRCs, remains challenging, a ‘test and treat’ POC strategy would help eliminate HCV in accordance with the objectives of the WHO.

## CONFLICT OF INTERESTS

XF acted as advisor for Gilead and AbbVie. JC had no conflict of interest. MGR received lecture fees from Gilead, AbbVie and Intercept and financial support for clinical projects (Gilead, AbbVie). JCQ conducted presentations in meetings and conferences (Gilead, AbbVie and MSD). (Grant of HCV microelimination Gilead‐AEEH 2021). SL acted as advisor for Gilead and AbbVie, speaker fees from Gilead, MSD and AbbVie. EM received lecture fees and research grants from Abbott GmbH & Co. K.G, Gilead Sciences, Cepheid and AbbVie and acted as advisor for Gilead Sciences. MB acted as advisor for Gilead and AbbVie. RDH and MAC are employees of Pharmacoeconomics & Outcomes Research Iberia, a consultancy firm specializing in the economic evaluation of healthcare interventions that have received unconditional funding from Gilead Sciences.

## Supporting information

Fig S1

Table S1

## Data Availability

The data supporting the findings of this study are available from the corresponding author XF upon reasonable request.
