# Supplementary material for: Point‐of‐care hepatitis C testing and treatment strategy for people attending harm reduction and addiction centres for hepatitis C elimination
Source: J Viral Hepat. 2021 Nov 29;29(3):227–30. doi: 10.1111/jvh.13634 (PMC9299793; doi:10.1111/jvh.13634)
Supplement: Supplementary file 1 — Fig S1 [file JVH-29-227-s001.pdf]

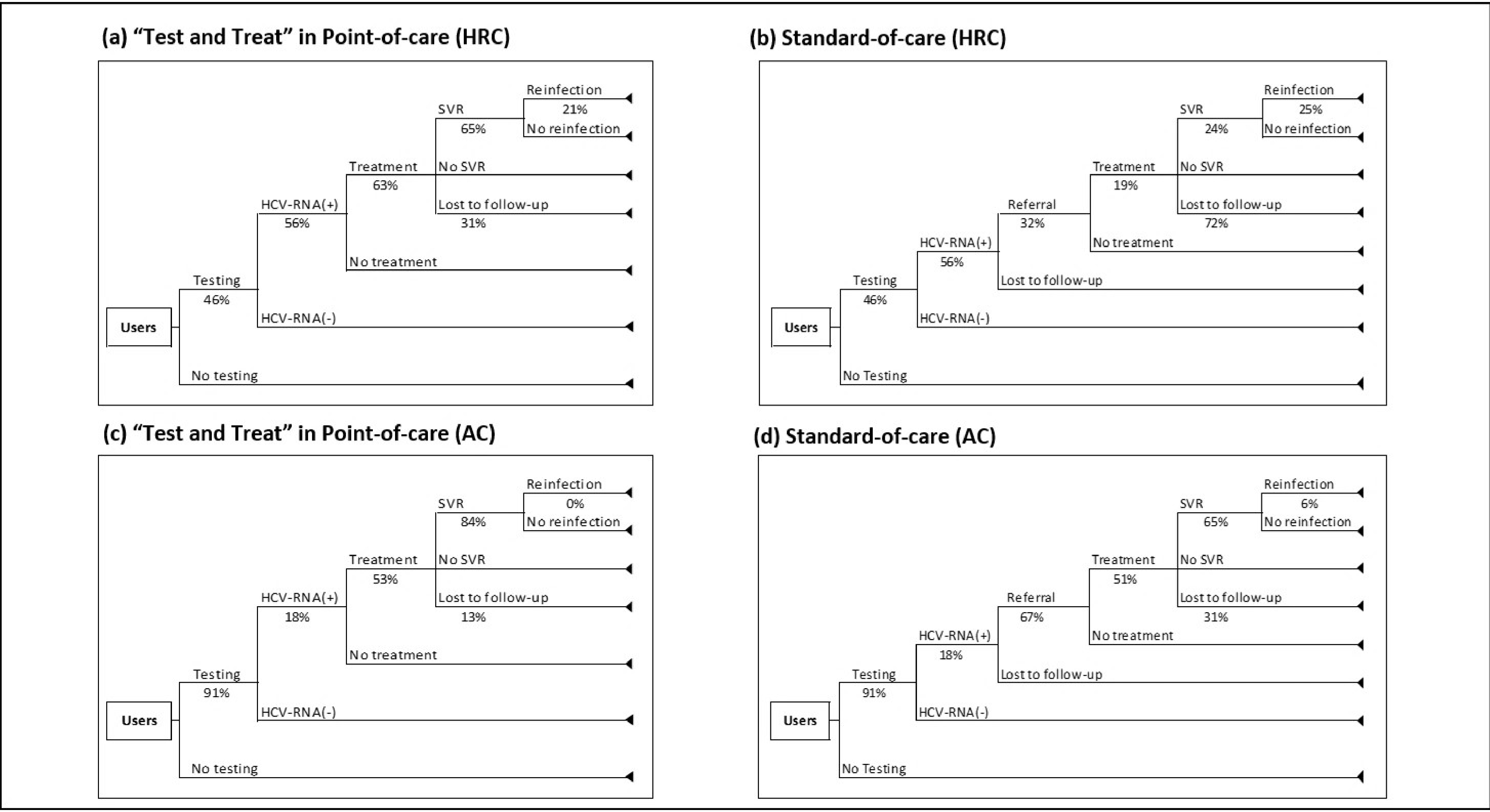
(a) "Test and Treat" in Point-of-care (HRC)
Users
Testing
46%
No testing
HCV-RNA(+)
56%
HCV-RNA(-)
Treatment
63%
No treatment
SVR
65%
No SVR
Lost to follow-up
31%
Reinfection
21%
No reinfection
(b) Standard-of-care (HRC)
Users
Testing
46%
No Testing
HCV-RNA(+)
56%
HCV-RNA(-)
Referral
32%
Lost to follow-up
Treatment
19%
No treatment
SVR
24%
No SVR
Lost to follow-up
72%
Reinfection
25%
No reinfection
(c) "Test and Treat" in Point-of-care (AC)
Users
Testing
91%
No testing
HCV-RNA(+)
18%
HCV-RNA(-)
Treatment
53%
No treatment
SVR
84%
No SVR
Lost to follow-up
13%
Reinfection
0%
No reinfection
(d) Standard-of-care (AC)
Users
Testing
91%
No Testing
HCV-RNA(+)
18%
HCV-RNA(-)
Referral
67%
Lost to follow-up
Treatment
51%
No treatment
SVR
65%
No SVR
Lost to follow-up
31%
Reinfection
6%
No reinfection
