# Supplementary material for: Point‐of‐care hepatitis C testing and treatment strategy for people attending harm reduction and addiction centres for hepatitis C elimination
Source: J Viral Hepat. 2021 Nov 29;29(3):227–30. doi: 10.1111/jvh.13634 (PMC9299793; doi:10.1111/jvh.13634)
Supplement: Supplementary file 2 — Table S1 [file JVH-29-227-s002.docx]

**Supplementary material**

**Table 1S. Analysis parameters**

| **Parameters** | **Value** | | **Reference** |
| --- | --- | --- | --- |
|  | **Point-of-care** | **Standard of care** |  |
| Harm Reduction Centres |  |  |  |
| Users | 6,878 | | [5] |
| Testing (HCV-RNA) | 46.2% | | [11] |
| Viremic infection (HCV-RNA+) | 55.5% | | [11] |
| Linkage-to-care | ----- | 31.9% | [6] |
| Treatment initiation | 62.6% | 18.6% | [6, 11] |
| SVR | 64.5% | 23.5% | [7, 11] |
| No SVR | 4.8% | 4.8% | [11]; assumption |
| Loss to follow-up | 30.7% | 71.7% | [11]; estimated percentage |
| Reinfection | 21.2% | 25.0% | [11] |
| Addiction Centres |  |  |  |
| Users | 13,778 | | [5] |
| Testing (HCV-RNA) | 91.2% | | [10] |
| Viremic infection (HCV-RNA+) | 17.9% | | [10] |
| Linkage-to-care | ----- | 67.2% | [8-10] |
| Treatment initiation | 53.4% | 51.1% | [8-10] |
| SVR | 84.1% | 65.2% | [8-10] |
| No SVR | 3.1% | 4.4% | [8-10] |
| Loss to follow-up | 12.9% | 30.5% | [8-10] |
| Reinfection | 0% | 5.9% | [10] |

SVR, sustained virologic response

**Figure 1S. Cascade of testing, linkage to care and treatment**

AC, addiction centre; HRD, harm reduction centre, SVR, sustained virological response
